# Supplementary material for: Mycobacterium tuberculosis Rv2387 Facilitates Mycobacterial Survival by Silencing TLR2/p38/JNK Signaling
Source: Pathogens. 2022 Aug 27;11(9):981. doi: 10.3390/pathogens11090981 (PMC9504853; doi:10.3390/pathogens11090981)
Supplement: Supplementary file 1 [file pathogens-11-00981-s001.zip › pathogens-1832252-supplementary.pdf]

Supplementary Material

Supplementary Materials and Methods

M. smegmatis phagocytosis assay

Microscopic visualization of *M. smegmatis* phagocytosis was performed as previously described [1]. Briefly, Msmg-2387 or Msmg-EV was stained with SYTO-9 (Thermo Scientific, USA) before infection. THP-1 or RAW 264.7 macrophages were then infected with SYTO-9-stained recombinant *M. smegmatis* at an MOI of 10. At 4 hrs post-infection, the macrophages were washed and fixed in a 4% paraformaldehyde solution for 0.5 hrs. Next, the macrophages were permeabilized with 0.2% (v/v) Triton X-100 solution for 20 min and stained with DAPI solution for 8 min in the dark. The images were obtained through the fluorescence microscope.

Analysis of the subcellular localization of Rv2387 protein in recombinant *M. smegmatis*

Subcellular fractionation was performed as previously described [2]. In brief, recombinant *M. smegmatis* strains were grown in medium at 37 °C to an OD_{600} of 0.6-0.8. Bacteria were harvested by centrifugation. Culture supernatants were filtered and concentrated 100-fold to obtain bacterial culture filtrate (CF). The whole cells were sonicated and centrifuged at 3,000 g for 30 min to obtain the whole cell lysates (WCL) from the supernatant. WCL was centrifuged at 27,000 g for 30 min. The pellet from this centrifugation step was considered the cell wall fraction (CW), and the supernatant was supposed to be the cell membrane and cytosolic (CM+Cy) fractions. All centrifugation steps were performed at 4 °C.

Equal amounts of protein from fractionated samples were subjected to Western blot analysis. The Myc-tagged Rv2387 protein was detected by an anti-Myc antibody (CTS).

GroEL, which served as a cytosol marker protein of mycobacteria [3], was detected by an anti-His primary antibody (CTS).

Supplementary Figures

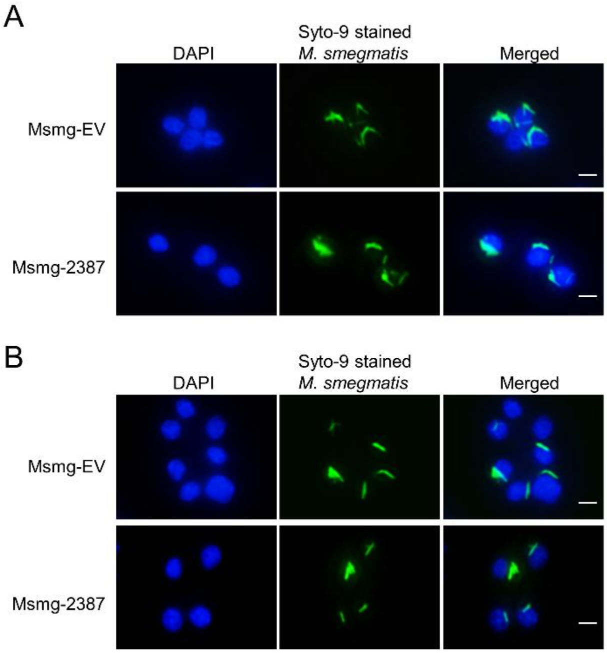

Figure S1. Microscopic visualization of *M. smegmatis* phagocytosis. THP-1 macrophages (A) or RAW 264.7 macrophages (B) were infected with SYTO-9-stained Msmg-2387 and Msmg-EV. Internalization of recombinant *M. smegmatis* by macrophages was visualized through a fluorescent microscope. The scale bars are equivalent to 5 μm. Similar results were obtained in two independent experiments.

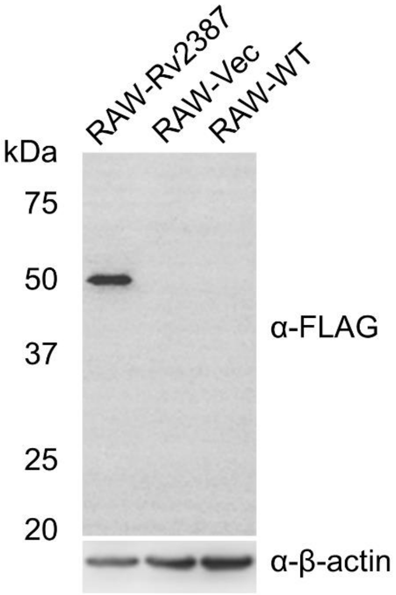

Figure S2. The expression of Rv2387 in RAW-Rv2387 cells. RAW-WT, RAW-vector, or RAW- Rv2387 cells were lysed in the sample buffer. Cell lysates were separated by SDS-PAGE, and proteins were detected with an anti-Flag antibody (Sigma).

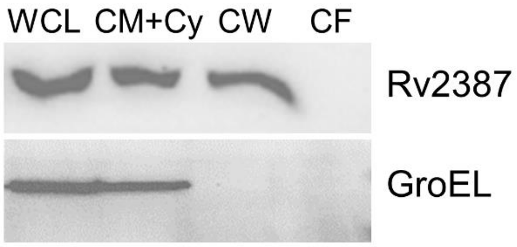

Figure S3. Subcellular localization of Rv2387 in MS_Rv2387. Equal amounts of protein from whole cell lysates (WCL), cytoplasmic membrane and cytoplasm (CM+Cy), cell wall (CW), and culture filtrate (CF) from MS_Rv2387 were separated by SDS-PAGE and proteins were detected with an anti-Myc antibody. Native GroEL was detected as a cytoplasmic control.

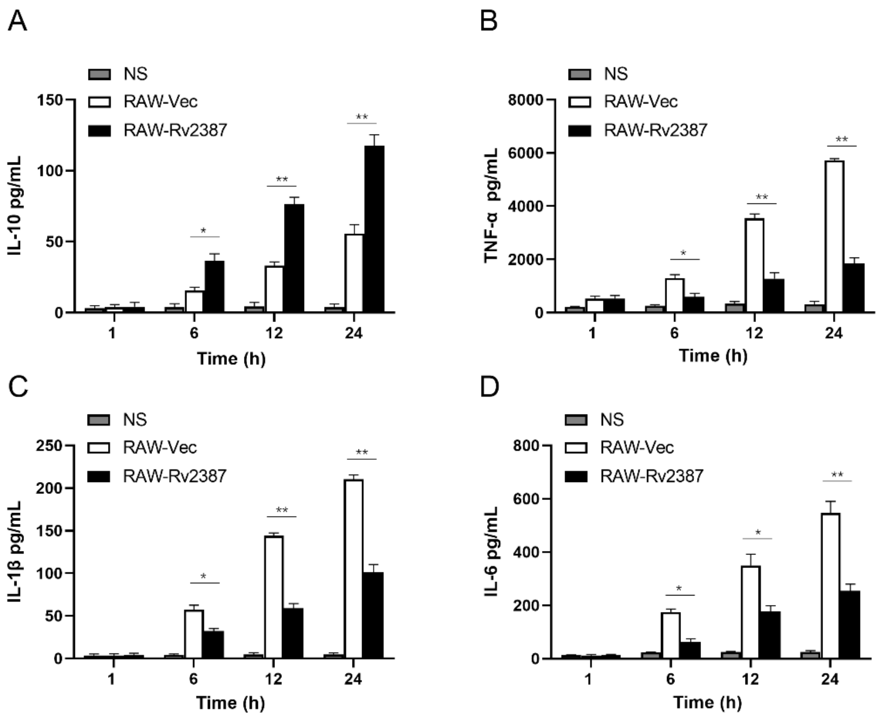

Figure S4. The expression of IL-10, TNF-α, IL-1β, and IL-6 in the RAW264.7 stable cells treated with TLR-2 agonist. RAW-Vec and RAW-Rv2387 macrophages were left unstimulated (NS) or treated with TLR2 agonist Pam3CSK4 (200 ng/mL). Culture supernatants were collected and subjected to an ELISA analysis to monitor the secretion of IL-10 (A), TNF-α (B), IL-1β (C), and IL-6 (D). Data are means ± SD of technical triplicate from one representative of two independent experiments. (* $p<0.05$, ** $p<0.01$).

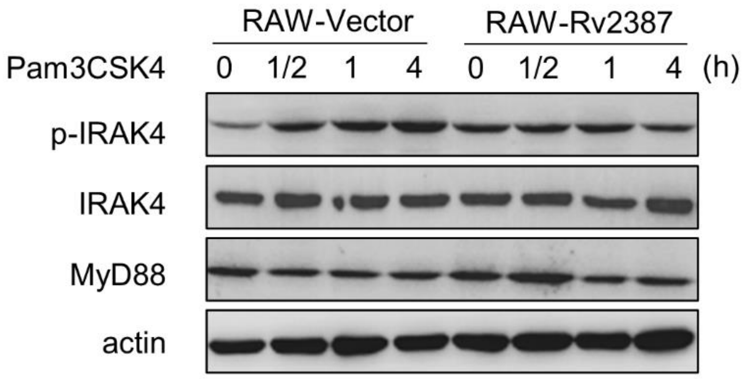

Figure S5. Rv2387 blocked the activation of IRAK4 in RAW-Rv2387 macrophages. Murine RAW-Vec and RAW-Rv2387 macrophages were treated with TLR2 agonist Pam3CSK4 (200 ng/mL) at different time points. Infected macrophages were lysed in RIPA buffer and subjected to immunoblot analysis to detect the protein expression of phosphorylated or total IRAK4, MyD88, and β-actin. Similar results were obtained in two independent experiments.

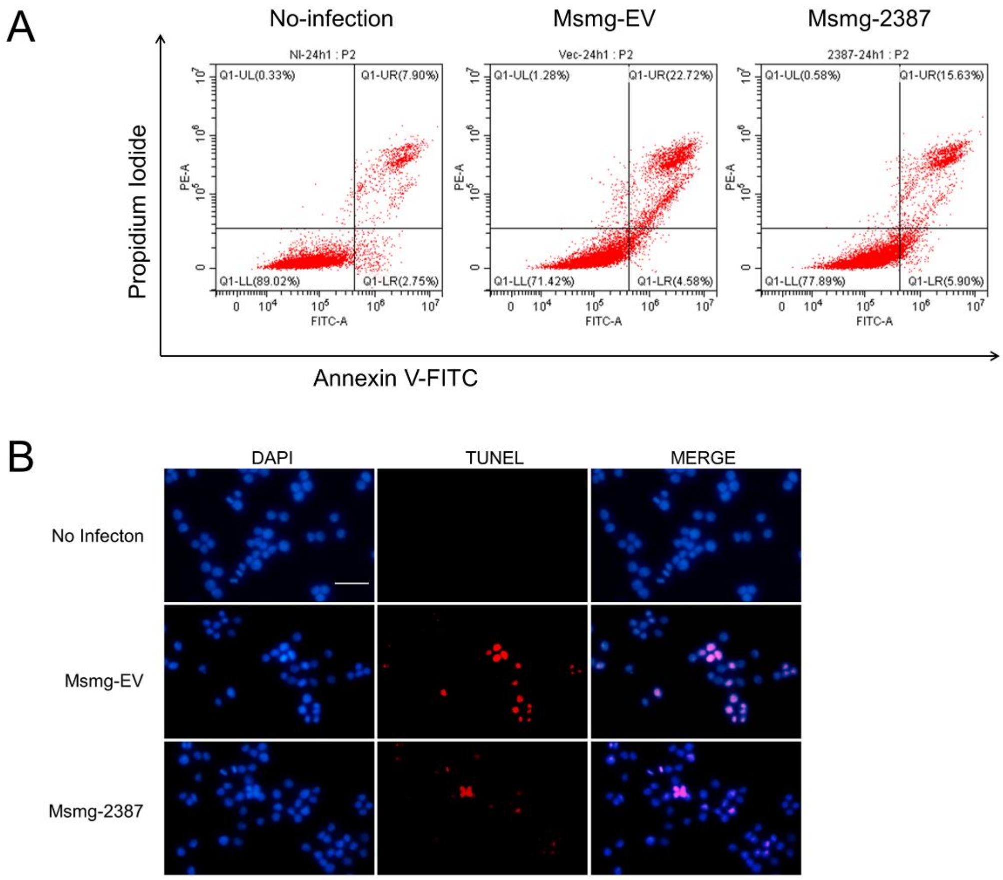

Figure S6. Rv2387 inhibited mycobacteria-induced macrophage apoptosis in BMDMs. A. a representative FACS profile corresponding to Fig. 7B. B. BMDMs were infected with either engineered Msmg-2387 or Msmg-EV strains for 24 hrs. DNA fragmentation in infected macrophages was detected via TUNEL staining analysis. The scale bars are equivalent to 50 μm. Similar results were obtained in two independent experiments.

Reference

1. Arora, S.K.; Naqvi, N.; Alam, A.; Ahmad, J.; Alsati, B.S.; Sheikh, J.A.; Kumar, P.; Mitra, D.K.; Rahman, S.A.; Hasnain, S.E.; et al. Mycobacterium smegmatis Bacteria Expressing *Mycobacterium tuberculosis*-Specific Rv1954A Induce Macrophage Activation and Modulate the Immune Response. *Front Cell Infect Microbiol.* **2020**. *10,* 564565.
2. Li, W.; Zhao, Q.; Deng, W.; Chen, T.; Liu, M.; Xie, J. *Mycobacterium tuberculosis* Rv3402c Enhances Mycobacterial Survival within Macrophages and Modulates the Host Pro-Inflammatory Cytokines Production via NF-Kappa B/ERK/p38 Signaling. *PLoS One* **2014**, *9*, e94418.
3. Bashiri, G., Perkowski, E.F.; Turner, A.P.; Feltcher, M.E.; Braunstein, M.; Baker, E.N. Tat-Dependent Translocation of an F(420)-Binding Protein of *Mycobacterium tuberculosis. PLoS One* **2012,** *7*, e45003.
